# Correlation Between Portal Pressure and Indocyanine Green Retention Rate is Unaffected by the Cause of Cirrhosis: A Prospective Study

**DOI:** 10.1007/s00268-021-06111-6

**Published:** 2021-04-23

**Authors:** Kosuke Kobayashi, Emilie Uldry, Takashi Kokudo, Alessandra Cristaudi, Yoshikuni Kawaguchi, Chikara Shirata, Takamune Yamaguchi, Olivier Dormond, Rafael Duran, Kiyoshi Hasegawa, Nicolas Demartines, Nermin Halkic

**Affiliations:** 1 Department of Visceral Surgery, Lausanne University Hospital and University of Lausanne, Rue du Bugnon 46, 1011 Lausanne, Switzerland; 2 Hepato-Pancreatico-Biliary Surgery Division, Department of Surgery, Graduate School of Medicine, The University of Tokyo, Tokyo, Japan; 3 Interventional Radiology, Lausanne University Hospital and University of Lausanne, Lausanne, Switzerland

## Abstract

**Background:**

Accurate estimation of the hepatic functional reserve before liver resection is important to avoid post-hepatectomy liver failure (PHLF). The aim of the present study was to evaluate the association of indocyanine green retention test with portal pressure by the cause of cirrhosis (non-viral vs. viral) and assessed postoperative outcomes including incidence of PHLF in patients with viral and non-viral cirrhosis.

**Methods:**

The cohort includes 50 consecutive patients with liver cirrhosis scheduled for liver resection for primary liver tumors at the Lausanne University Hospital between 2009 and 2018.

**Results:**

There were 31 patients with non-viral liver cirrhosis (Non-virus group) and 19 with viral liver cirrhosis (virus group). The indocyanine green retention rate at 15 min (ICG-R15) (*p* = 0.276), Hepatic Venous Portal Gradient (HVPG; *p* = 0.301), and postoperative outcomes did not differ between the non-virus group and viral group. ICG-R15 and HVPG showed a significant linear correlation in all patients (Spearman’s rank correlation coefficient, *ρ* = 0.599, *p* < 0.001), the non-virus group (*ρ* = 0.555, *p* = 0.026), and the virus group (*ρ* = 0.534, *p* = 0.007). A receiver operating characteristic curve analysis showed that ICG-R15 was a predictor for presence of portal hypertension (PH; HVPG ≥ 12 mmHg) (area under the curve [AUC] = 0.780). The cut-off value of ICG-R15 for predicting the presence of PH was 16.0% with 72.3% of sensitivity and 79.0% of specificity.

**Conclusions:**

The ICG-R15 level was associated with portal pressure in both patients with non-virus cirrhosis and patients with virus cirrhosis and predicts the incidence of PH with relatively good discriminatory ability.

**Clinical trial number:**

https://clinicalTrials.gov(ID:NCT00827723)

**Local ethics committee number:**

CER-VD 251.08

## Introduction

Post-hepatectomy liver failure (PHLF) is a severe complication associated with the high mortality rate [1, 2]. Accurate estimation of the hepatic functional reserve before liver resection is important to avoid PHLF [3]. Portal hypertension (PH) is a known factor associated with liver cirrhosis and poor liver function. As such, in Europe and the US, PH was a contraindication for liver resection according to the previous edition of Barcelona Clinic of Liver Cancer (BCLC) guidelines [4]. Recent studies and guidelines suggested that hepatectomy for patients with PH should not be a contraindication. Nonetheless, PH remains an important factor for prognosis [1, 5, 6]. In contrast, PH is not a contraindication for liver resection in Asia, where indocyanine green retention rate at 15 min (ICG-R15) is widely used to evaluate hepatic functional reserve for avoiding PHLF [7–9]. For evaluating PH, the portal pressure is measured; however, it is more invasive and complex than the evaluation of ICG-R15.

Wadhawan et al. [10] reported that patients with alcoholic cirrhosis tended to have higher portal pressure than those with viral cirrhosis and similar hepatic function reserve. We hypothesized that ICG-R15 estimates the degree of portal hypertension in patients with non-viral and viral cirrhosis and can be used as an indicator to avoid PHLF. To address these issues, we evaluated the association of ICG-R15 with portal pressure by the cause of cirrhosis (non-viral vs. viral) and assessed postoperative outcomes including incidence of PHLF in patients with viral and non-viral cirrhosis.

## Methods

This study was approved by the local ethics committee (registration number CER-VD 251.08) and was registered at https://clinicalTrials.gov (ID: NCT00827723). All patients provided written informed consent for the study.

### Study design

This was a prospective observational study to assess the association of ICG-R15 with preoperative portal pressure in patients with viral and non-viral cirrhosis and primary liver cancer. Liver cirrhosis was diagnosed by liver biopsy at our institution or other hospitals. Patients were enrolled from January 2009 through December 2018 at the Lausanne University Hospital. ICG retention rate and portal pressure were measured on the same day (one week before surgery) in all patients.

### ICG retention test

Patients fasted from midnight before the morning for ICG retention test. Peripheral intravenous lines were placed in both forearms. ICG was injected rapidly for 30 s into one arm at a dose of 0.5 mg/kg of ICG (Verdye^®^, Diagnostic Green, Aschheim-Dornach, Germany) diluted to a concentration of 5 mg/mL with distilled water. Blood samples were taken from another arm 5, 10 and 15 min after the injection. After centrifugation, the samples were analyzed by spectrophotometry at a wavelength of 805 nm. The value of ICG-R15 was assessed with the calibration curves as described elsewhere [11].

### Measurement of portal pressure

Portal pressure was measured on the basis of the hemodynamic procedure described by Groszmann et al. [12]. After using local anesthesia, Seldinger technique was used to insert an introducer into the right internal jugular vein. A 5Fr of balloon catheter was placed into the right hepatic vein under fluoroscopic vision. The balloon was inflated to measure the wedged hepatic vein pressure (WHVP), which corresponds to the sinusoidal portal vein pressure. The balloon was deflated to measure the free hepatic venous pressure (FHVP), which corresponds to the pressure of the inferior vena cava. The veno–venous gradient (Hepatic venous portal gradient = HVPG) was calculated as follows: HVPG = WHVP − FHVP. A HVPG ≥ 12 mmHg was defined as having PH [13, 14].

### Preoperative management

Preoperative evaluation included routine clinical and laboratory tests (hematology, biochemistry, liver function and coagulation tests, tumor markers), CT volumetry to determine the surgical procedures, indications for portal vein embolization, and future liver remnant volume. Surgical procedures were selected on the basis of preoperative findings of primary liver tumor. The extent of liver resection was determined under the Makuuchi’s criteria [7].

### Postoperative surveillance

Postoperative complications were categorized using the Clavien classification [15] and comprehensive complication index (CCI) [16]. Patients were followed one month and 6 months after surgery (including imaging assessment with CT or ultrasound), and then followed twice a year, either at our hospital or at clinics of primary care physicians. Recurrence was diagnosed on the basis of imaging findings, clinical data, and/or histopathological studies.

### Definitions

Model for End-stage Liver Disease (MELD) scores were calculated using serum bilirubin, serum creatinine, and PT-INR as follows [17]: 9.57 × loge (creatinine [mg/dL]) + 3.78 × loge (total bilirubin [mg/dL]) + 11.2 × loge (INR) + 6.43. The Albumin–Indocyanine Green evaluation (ALICE) scores were calculated as follows [18]: 0.663 × log10 (ICG R15 [%]) − 0.0718 × (albumin [g/dL]). ALICE grades were determined according to ALICE scores as follows [18]: grade 1, − 2.20 or less; grade 2a, − 2.20 to − 1.88 or less; grade 2b, − 1.88 to − 1.39 or less, and grade 3, greater than − 1.39. Surgical complexity of liver resection procedures was stratified using the three-level complexity classification, which classifies 11 different liver resection procedures as grade I (low complexity; wedge resection and left lateral sectionectomy), grade II (intermediate complexity; anterolateral segmentectomy and left hepatectomy) or grade III (high complexity; posterosuperior segmentectomy, right posterior sectionectomy, right hepatectomy, central hepatectomy, and extended left/right hepatectomy) [19–22]. The PHLF was defined according to the International Study Group of Liver Surgery (ISGLS) criteria [23].

### Statistical analysis

Categorical variables are expressed as n (%) and were compared between groups using Fisher’s exact test or the *χ*^2^ test as appropriate. Continuous variables are expressed as median (interquartile range [IQR]) and were compared using Wilcoxon’s rank test. CCI scores were expressed as mean (standard deviation [SD]), and were compared among groups using the ANOVA test for three groups. Correlations between independent variables were determined using Spearman’s rank correlation test. The ICG-R15 and portal pressure were evaluated using the receiver operating characteristic (ROC) curve analysis, and area under the curve (AUCs) was compared to evaluate the predictor for the presence of PH. A *p* value < 0.05 was considered as statistical significance. All statistical analysis was performed using JMP 13.2.0 software (SAS Institute, Cary, NC, USA).

## Results

**Fig. 1:**
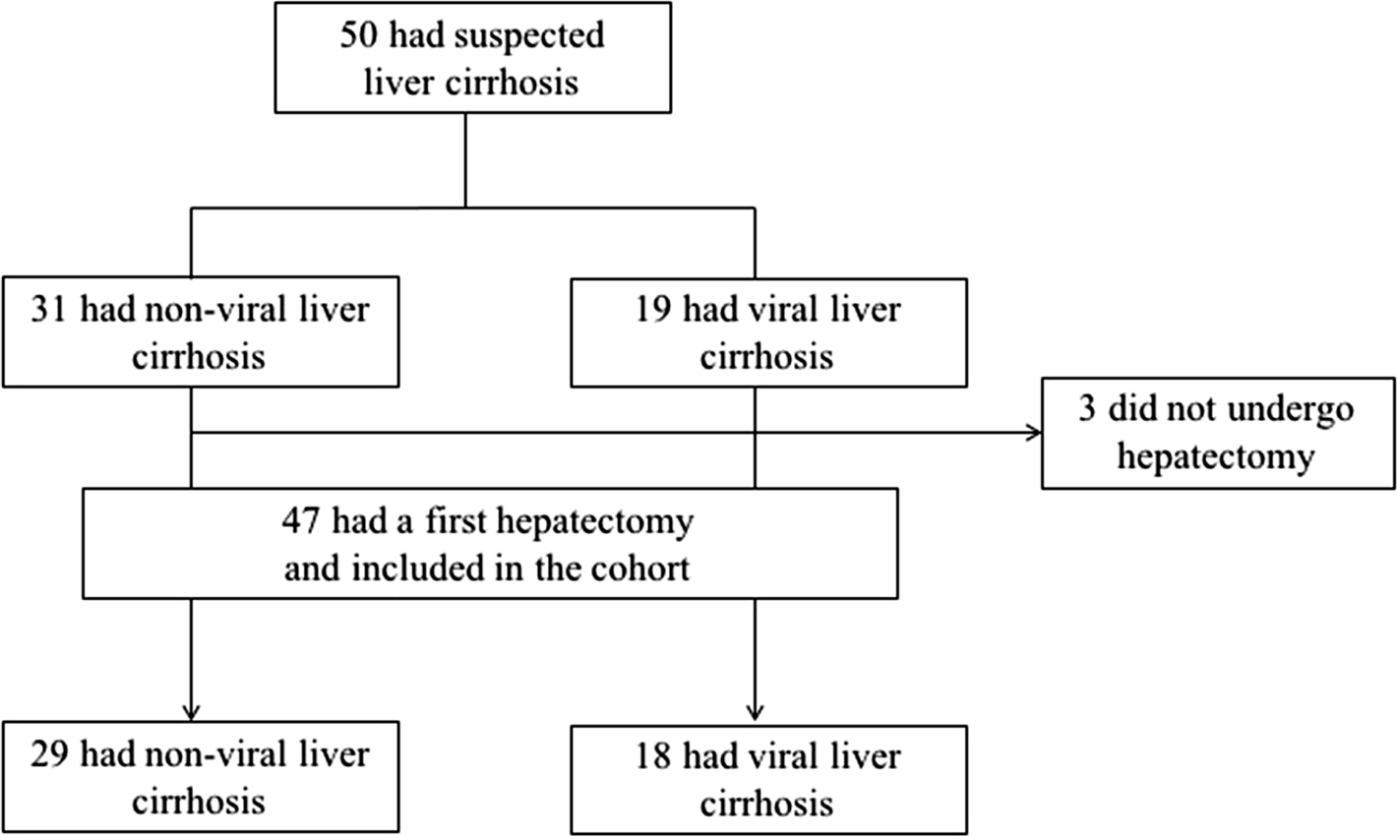
Patient selection

During the study period, we enrolled 50 patients. Of these, 31 patients had non-virus liver cirrhosis (the non-virus group) including alcoholic liver cirrhosis (*n* = 22) and non-alcoholic steatohepatitis (*n* = 9) (Fig. 1). Of the non-virus group, 29 patients (93.5%) underwent hepatectomy. The remaining 19 patients had viral liver cirrhosis (the virus group), and 18 patients underwent liver resection (Fig. 1). Of the 50 patients, 3 patients were excluded from the study because 2 patients underwent trans-arterial chemoembolization and one patient underwent radioembolization because tumor progressed or liver function became worse to facilitate liver resection.

### Clinical characteristics

Patients’ demographics were summarized in Table 1. The virus group included 18 patients with hepatocellular carcinoma (HCC) (94.7%) and one with cholangiocarcinoma (5.3%). The non-virus group included 25 patients with HCC (80.7%), 4 with cholangiocarcinoma (12.9%), and 2 with lymphoma (6.5%). The median age and median body mass index were significantly higher in the non-virus group than in the virus group: median (IQR) age, 66 (64–71) years versus 60 (44–71) years, *p* = 0.044; median (IQR) body mass index, 27.5 kg/m^2^ versus 23.6 kg/m^2^, *p* = 0.00). The rate of American Society of Anesthesiologists physical status classification ≥ 3 was significantly higher in the non-virus than in the virus group (60.0% vs. 16.7%, *p* = 0.003). Between the non-virus group and the virus group, ICG-R15 and HVPG, and presence of PH did not differ significantly: ICG-R15, 15.0% (IQR, 9.0–19.3) versus 12.1% (IQR, 5.0–15.0), *p* = 0.276; median HVPG, 8 mmHg (IQR, 5–12) versus 7 mmHg (IQR, 4–10), *p* = 0.301; the presence of PH, 25.8% versus 15.8%, *p* = 0.329. Surgical complexity did not differ significantly between the groups.

**Table 1:** Baseline characteristics and Portal pressure measurement

Variables	Non-virus group	Viral group	*P* value
	*n* = 31	*n* = 19	
Age, year	66 (64–71)	60 (44–71)	0.044
Sex, male: female	29:2	14:5	0.052
BMI, kg/m^2^	27.5 (24.5–29.3)	23.6 (20.5–24.9)	0.001
ASA PS classification > 3	18 (60.0%)	3 (16.7%)	0.003
Etiology of virus, HBV/HCV/HBV + HCV	–	8/8/2	
Liver cirrhosis	31 (100%)	19 (100%)	> 0.999
Albumin, g/l	41 (38–44)	42 (38–45)	0.756
Total bilirubin, µmol/l	10 (10–12)	10 (5–14)	0.738
Platelet, *10^9^/l	205 (160–240)	187 (137–222)	0.319
PT, %	90 (80–100)	100 (85–100)	0.144
INR	1.1 (1.0–1.1)	1.0 (1.0–1.1)	0.072
Creatinin, µmol/l	86 (67–96)	85 (69–96)	0.787
Child–Pugh score			
Class *A*/*B*/*C*	2/ 29/ 0	1/ 18/ 0	0.864
MELD score	9 (7–10)	8 (5–9)	0.234
ALICE score	− 2.15 (− 2.56 to − 1.90)	− 2.22 (− 2.66 to − 1.95)	0.448
Grade 1	15 (50.0%)	10 (52.6%)	
Grade 2a	8 (26.7%)	5 (26.3%)	
Grade 2b	6 (20.0%)	3 (15.8%)	
Grade 3	1 (3.3%)	1 (5.3%)	
ICG-R15, %	15.0 (9.0–19.3)	12.1 (5.0–15.0)	0.276
Portal pressure measurement			
WHVP, mmHg	16 (9–19)	13 (10–17)	0.105
FHVP, mmHg	8 (7–11)	7 (5–10)	0.426
HVPG, mmHg	8 (5–12)	7 (4–10)	0.301
Presence of portal hypertension	8 (25.8%)	3 (15.8%)	0.329
Diagnosis			
Hepatocellular carcinoma	25 (80.7%)	18 (94.7%)	0.163
Cholangiocarcinoma	4 (12.9%)	1 (5.3%)	0.382
Lymphoma	2 (6.5%)	0	0.259

Data are presented as median (IQR) or *n* (%)

*BMI* body mass index; *ASA PS classification* American Society of Anesthesiologists physical status classification; *PT* prothrombin time; *ALICE* Albumin–Indocyanine green evaluation; *ICG-R15* Indocyanine green retention test at 15-min; *WHVP* Venital Pressure Pressure; *FHVP* Free hepatic venous pressure; *HVPG* Hepatic venous portal gradient

### Comparison of intra- and postoperative outcomes by groups

The intra- and postoperative outcomes are shown in Table 2. There were no significant differences in perioperative outcomes, liver enzymes concentrations, or morbidity between the groups. Two mortality occurred because of PHLF: one patient without PH in the viral group developed partial segmental devascularization and subsequent severe infection, and another patient with PH in the non-virus group developed postoperative hemorrhage.

**Table 2:** Intra- and post- operative outcomes

Variables	Non-virus group *n* = 29	Virous group *n *= 18	*P* value
*Intraoperative outcomes*			
Surgical complexity			0.590
Grade I	4 (12.8%)	4 (22.2%)	
Grade II	10 (34.5%)	4 (22.2%)	
Grade III	15 (51.7%)	10 (55.6%)	
Laparoscopic approach	5 (17.3%)	6 (33.3%)	0.205
Estimated blood loss, ml	600 (275–953)	700 (288–1075)	0.956
Estimated blood loss ≥ 500 ml	20 (55.6%)	10 (66.7%)	0.441
RBC transfusion	6 (20.7%)	3 (16.7%)	0.733
5-days after operation			
Albumin, g/l	34 (31–39)	38 (30–43)	0.196
Total bilirubin, µmol/l	13 (10–22)	13 (10–29)	0.991
PT, %	85 (70–100)	90 (73–110)	0.285
INR	1.1 (1.0–1.2)	1.0 (1.0–1.1)	0.329
Total bilirubin maximum, µmol/l	26 (17–34)	19 (12–46)	0.490
PT minimum, %	60 (55–70)	63 (55–85)	0.265
INR maximum	1.3 (1.2–1.4)	1.2 (1.1–1.3)	0.186
*Postoperative morbidity*			
Morbidity	18 (62.1%)	11 (61.1%)	0.948
Clavien-Dindo classification ≥ III	3 (11.1%)	4 (25.0%)	0.233
Comprehensive complication index	8.7 (0–23.6)	8.7 (0–26.2)	0.901
ISGLS B/C	10 (33.3%)	4 (22.2%)	0.412
Mortality	1 (3.5%)	1 (5.6%)	0.728
Postoperative length of stay, days	9 (6–16)	9 (7–19)	0.948

Data are presented as median (IQR) or *n* (%)

*RBC* red blood cell; *PT* prothrombin time; *ISGLS* The posthepatectomy liver failure defined by the International Study Group of Liver Surgery

### Correlation between ICG-R15 and HVPG

**Fig. 2:**
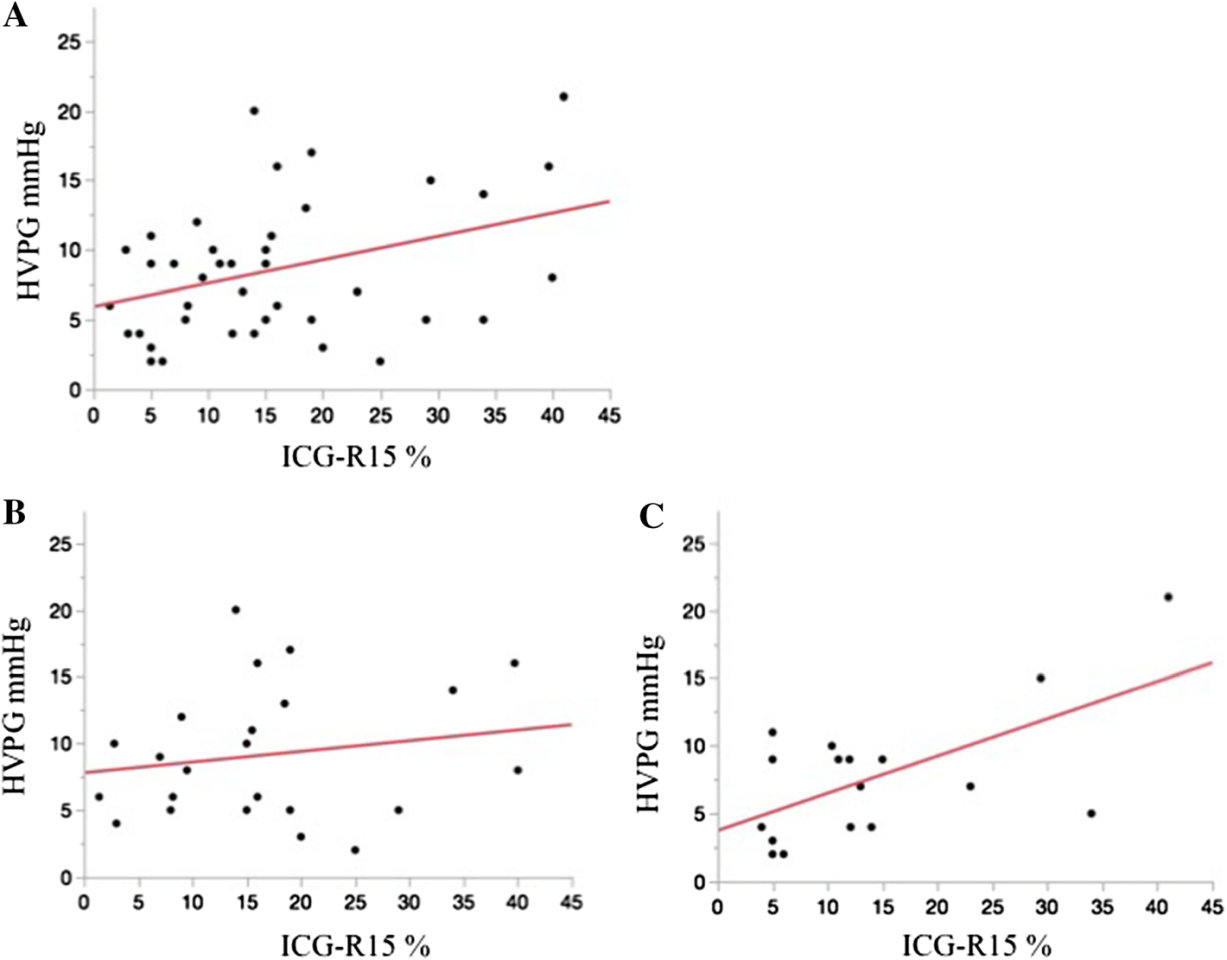
Correlation between the indocyanine green retention rate at 15 min (ICG-R15) and Hepatic Venous Portal Gradient (HVPG) A linear correlation was observed between ICG-R15 and HVPG. Spearman’s rank correlation coefficient of ICG-R15 and HVBG showed a significant linear correlation (**a**) in all patients (*ρ* = 0.599, *p* < 0.001), **b** the non-virus group (*ρ* = 0.555, *p* = 0.026), (**c**) and the virus group (*ρ* = 0.534, *p* = 0.007)

ICG-R15 and HVPG showed a significant linear correlation in all patients (Spearman’s rank correlation coeffient, *ρ* = 0.599, *p* < 0.001), the non-virus group (*ρ* = 0.555, *p* = 0.026), and the virus group (*ρ* = 0.534, *p* = 0.007) (Fig. 2).

### Performance of ICG-R15 for predicting presence of PH and PHLF

**Fig. 3:**
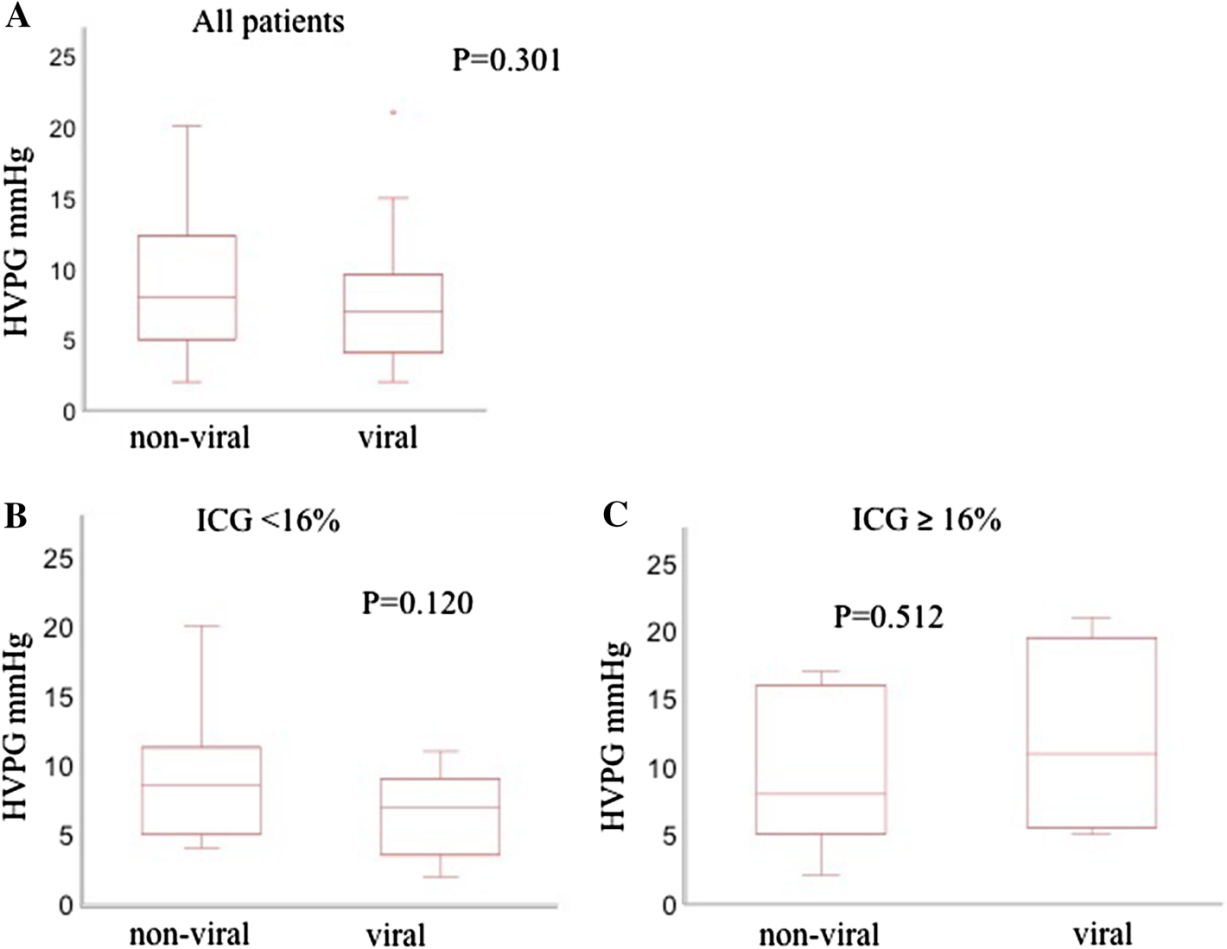
Portal pressure between non-virus group and virus group in all patients (**a**), patients with the indocyanine green retention rate < 16.0% (**b**), and in patients with the indocyanine green retention rate ≥ 16% (**c**)

A receiver operating characteristic (ROC) curve analysis showed that ICG-R15 was a predictor for presence of PH (area under the curve [AUC] = 0.780). The cut-off value of ICG-R15 for predicting the presence of PH was 16.0% with 72.3% of sensitivity and 79.0% of specificity. The portal pressure did not differ significantly between non-virus group and virus group when hepatic functional reserve was stratified by the ICG-R15 level (< 16.0% or ≥ 16%) (Fig. 3).

### Outcomes of patients with or without PH

**Fig. 4:**
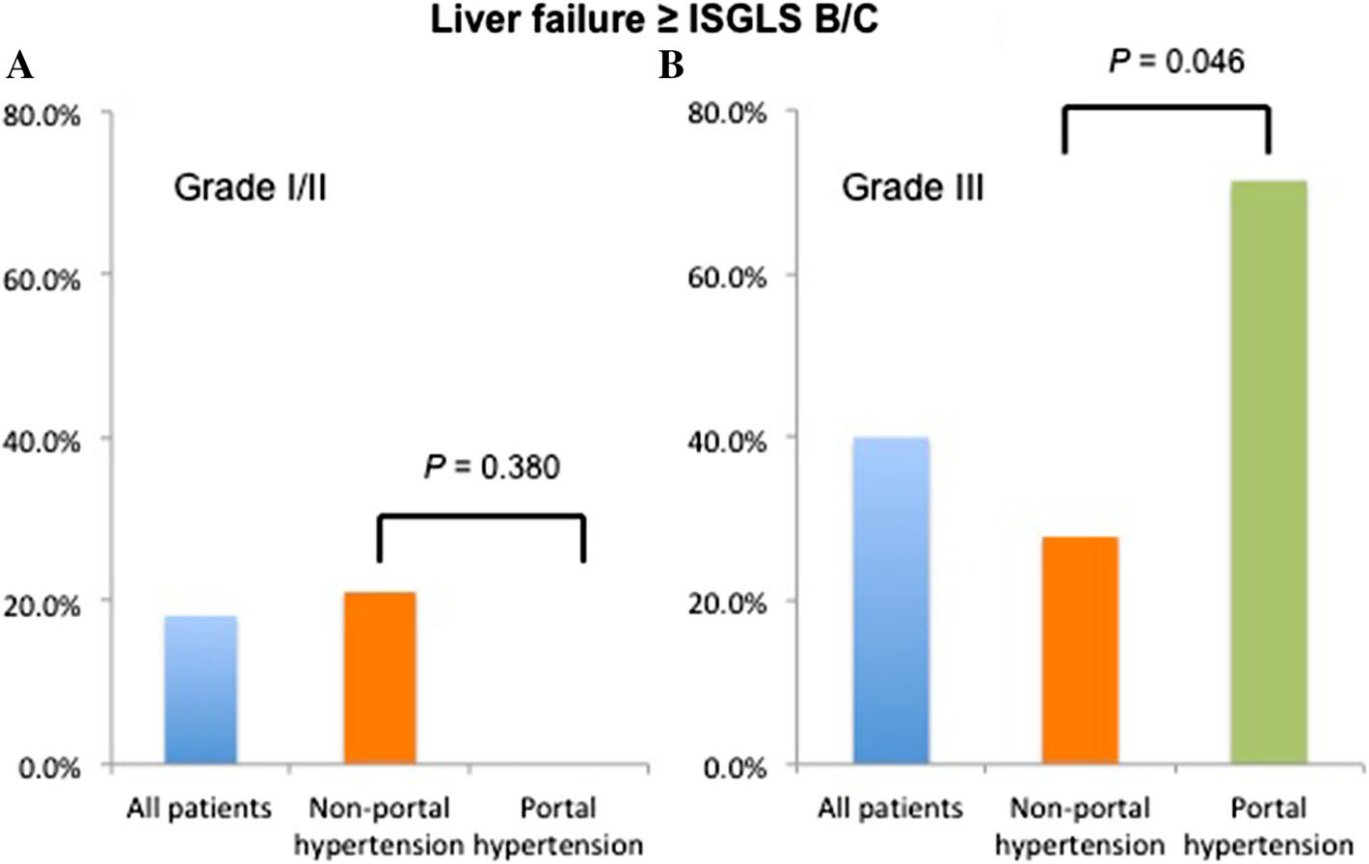
Liver failure rate stratified by the three-level complexity classification in patients undergoing resection of grade I/II procedures (**a**) and patients undergoing resection of grade III procedures (**b**)

The ICG-R15 level was significantly lower in patients without PH than in patients with PH (median 12.0% [IQR: 6.8–15.2] versus 19.0% [IQR: 14.0–39.7], *p* = 0.005). The rates of major complication (Clavien classification ≥ 3) (22.2% vs. 20.0%, *p* = 0.880) and liver failure (ISGLS grade ≥ B) (23.7% vs. 50.0%, *p* = 0.103) did not differ significantly between both groups (Table 3). Postoperative liver failure was further assessed with the stratification of the three-level complexity classification and the presence of PH (Fig. 4). In patients underwent resection of grade III procedures, the rate of liver failure (ISGLS grade ≥ B) was significantly higher in patients with PH than in patients without PH (71.4% vs. 27.8% *p* = 0.046), whereas in patients undergoing resection of grade I or II procedures, the rate of liver failure did not differ significantly between the groups (0% vs. 21.1%).

**Table 3:** Demographics, intraoperative, and postoperative outcomes by presence of portal hypertension (HVPF ≥ 12 mmHg)

Variables	PH group *n* = 11	Non-PH group *n* = 39	*P* value
Demographics			
Age, year	65 (61–75)	66 (60–70)	0.648
Non-virus cirrhosis: viral cirrhosis	2:9	17:22	0.125
Albumin, g/l	37 (33–42)	42 (39–45)	0.004
Total bilirubin, µmol/l	14 (11–18)	10 (6–11)	0.001
Platelet, *10^9^/l	169 (129–223)	206 (160–238)	0.256
PT, %	80 (65–100)	100 (80–100)	0.534
ALICE score	− 1.681 (− 2.000 to 1.521)	− 2.322 (− 2.631 to − 2.084)	0.001
ICG-R15, %	19.0 (14.0–39.7)	12 (6.8–15.2)	0.005
Intraoperative outcomes	*n* = 10	*n* = 37	
Surgical complexity			0.484
Grade I	1 (10.0%)	7 (18.9%)	
Grade II	2 (20.0%)	12 (32.4%)	
Grade III	7 (70.0%)	18 (48.7%)	
Laparoscopic approach	1 (10.0%)	10 (27.0%)	0.275
Estimated blood loss, ml	850 (575–2025)	500 (200–925)	0.032
Estimated blood loss ≥ 500 ml	9 (90.0%)	21 (56.8%)	0.044
RBC Transfusion	4 (40.0%)	5 (13.5%)	0.053
5-days after operation			
Albumin, g/l	32 (30–36)	36 (31–40)	0.091
Total bilirubin, µmol/l	22 (12–60)	11 (10–19)	0.031
PT, %	75 (60–95)	90 (75–100)	0.208
Total bilirubin maximum, µmol/l	39 (21–72)	20 (13–29)	0.042
PT minimum, %	55 (52–65)	65 (55–73)	0.125
Postoperative morbidity			
Morbidity	10 (100%)	19 (51.4%)	0.008
Clavien-Dindo classification ≥ III	2 (20.0%)	8 (22.2%)	0.880
Comprehensive complication index	20.9 (20.9–40.1)	8.7 (0–23.6)	0.004
ISGLS *B*/*C*	5 (50.0%)	9 (23.7%)	0.103
Mortality	1 (10.0%)	1 (2.7%)	0.310
Postoperative length of stay, days	14 (10–19)	8 (6–15)	0.046

Data are presented as median (IQR) or *n* (%)

*HVPG* Hepatic Venous Portal Gradient; *PT* prothrombin time; *ALICE* Albumin–Indocyanine green evaluation; *ICG-R15* Indocyanine green retention test at 15-min; *RBC* red blood cell; *PT* prothrombin time; *ISGLS* The posthepatectomy liver failure defined by the International Study Group of Liver Surgery

## Discussion

The findings of the study showed that the ICG-R15 level was correlated with portal pressure irrespective of the cause of cirrhosis (i.e., non-virus cirrhosis and virus cirrhosis). The ICG-R15 level and the three-level complexity classification were associated with the incidence of PHLF. As such, the test of ICG-R15 can be used to estimate portal pressure as a less-invasive method and to predict the incidence of PHLF as did the three-level complexity classification.

An earlier study showed that portal pressure was higher in patients with non-virus alcoholic cirrhosis than in patients with virus cirrhosis when compared in patients who had similar hepatic functional reserve [10]. In contrast, our study showed that portal pressure did not differ significantly between patients with non-virus cirrhosis and those with virus cirrhosis when hepatic functional reserve was stratified by the ICG-R15 level (Fig. 3). The ROC curve analysis in our study showed that the cut-off value for effectively predicting the presence of PH was ICG-R15 ≥ 16.0%. This was in line with a previous study which showed that ICG-R15 is an accurate noninvasive method to identify clinically relevant PH and the cut-off value for effectively predicting the presence of PH was ICG-R15 ≥ 16.7% [24].

Our study showed that in patient undergoing grade III (high complexity) resection, liver failure (ISGLS grade ≥ B) developed more frequently in patients with PH than in patients without PH, whereas in patients undergoing grade I and II (low/intermediate complexity) resections, the rate of liver failure did not differ significantly between the groups. Specifically, we showed that liver resection of low/intermediate complexity grade procedures can be safely performed with low risk of PHLF. Advancements in surgical techniques and perioperative management have led liver resection safer and decreased postoperative mortality rate [25–28]. Although the BCLC Staging System and treatment guidelines recommends that PH is considered a contraindication to liver resection [29, 30], a recent systematic review and meta-analysis showed that the presence of PH should not be regarded as an absolute contraindication to surgery, whereas PH is a prognostic factor for survival [5]. Additionally, studies reported that liver resection is routinely performed in patients with HCC and PH in specialized centers worldwide [31–35]. The BCLC Staging System and treatment guidelines with respect to the contraindication of resection for patients with PH was based on a study which compared patients who had PH with patients who did not have PH [4]. However, the study included only 6 patients undergoing wedge resection and 23 patients undergoing anatomical resection. This suggested that the study may have included patients who underwent resection of high complexity grade procedures. Our study implied that liver resection of low/intermediate complexity grade procedures can be safely performed in patients with cirrhosis and PH. Recent European Association for the Study of the Liver Clinical Practice Guidelines suggested that the risk of minor hepatectomy in patient with cirrhosis and PH was intermediate and comparable to the risk of major hepatectomy in patients without PH [1, 36].

The major limitation of the present study was the small number of patients. During the period more than 10 years, only 50 patients were accrued. This was due to the low incidence of HCC in Europe. Nonetheless, ours is a first study to compare the association of ICG-R15 with PH in patients with or without virus cirrhosis and showed the usefulness of ICG-R15 and the three-level complexity classification to predict PHLF.

## Conclusions

In conclusion, the ICG-R15 level was associated with portal pressure in both patients with non-virus cirrhosis and patients with virus cirrhosis and predicts the incidence of PH with relatively good discriminatory ability. The ICG-R15 and surgical complexity were predictors for PHLF and might be able to use to decide the surgical indication in patients with PH.

## Abbreviations

PHLF: Post-hepatectomy liver failure
BCLC: The barcelona clinic of liver cancer
PH: Portal hypertension
ICG-R15: Indocyanine green retention rate at 15 min
MELD: Model for end-stage liver disease
ALICE: Albumin–indocyanine green evaluation
WHVP: Wedged hepatic vein pressure
FHVP: Free hepatic venous pressure
HVPG: Hepatic venous portal gradient
SPH: Severe portal hypertension
IQR: Interquartile range
ROC: Receiver operating characteristic
AUC: Area under the curve
ASA PS classification: American society of anesthesiologists physical status classification
